# Using theories of practice to understand HIV-positive persons varied engagement with HIV services: a qualitative study in six Sub-Saharan African countries

**DOI:** 10.1136/sextrans-2016-052977

**Published:** 2017-07-23

**Authors:** Morten Skovdal, Alison Wringe, Janet Seeley, Jenny Renju, Sara Paparini, Joyce Wamoyi, Mosa Moshabela, William Ddaaki, Constance Nyamukapa, Kenneth Ondenge, Sarah Bernays, Oliver Bonnington

**Affiliations:** 1 Department of Public Health, University of Copenhagen, Copenhagen, Denmark; 2 Biomedical Research and Training Institute, Harare, Zimbabwe; 3 London School of Hygiene and Tropical Medicine, London, UK; 4 Africa Health Research Institute, KwaZulu-Natal, South Africa; 5 Medical Research Council/Uganda Virus Research Institute Research Unit on AIDS, Entebbe, Uganda; 6 Malawi Epidemiology and Intervention Research Unit, Karonga, Malawi; 7 National Institute for Medical Research, Mwanza, Tanzania; 8 University of KwaZulu Natal, Durban, South Africa; 9 Rakai Health Sciences Program, Kalisizo, Uganda; 10 Imperial College London, London, UK; 11 Kenya Medical Research Institute, Kisumu, Kenya

**Keywords:** Social Theory, Patient Engagement, HIV, Health Services Research, Highly Active Antiretroviral Therapy, Africa

## Abstract

**Objectives:**

This article considers the potential of ‘theories of practice’ for studying and understanding varied (dis)engagement with HIV care and treatment services and begins to unpack the assemblage of elements and practices that shape the nature and duration of individuals’ interactions with HIV services.

**Methods:**

We obtained data from a multicountry qualitative study that explores the use of HIV care and treatment services, with a focus on examining the social organisation of engagement with care as a practice and as manifested in the lives of people living with HIV in sub-Saharan Africa. The dataset comprised of 356 interviews with participants from six countries.

**Results:**

We noted fluctuating interactions with HIV services in all countries. In line with theories of practice, we found that such varied engagement can be explained by (1) the availability, absence and connections between requisite ‘materialities’ (eg, health infrastructure, medicines), ‘competencies’ (eg, knowing how to live with HIV) and ‘meanings’ (eg, trust in HIV services, stigma, normalisation of HIV) and (2) a host of other life practices, such as working or parenting. These dynamics either facilitated or inhibited engagement with HIV services and were intrinsically linked to the discursive, cultural, political and economic fabric of the participating countries.

**Conclusion:**

Practice theory provides HIV researchers and practitioners with a useful vocabulary and analytical tools to understand and steer people’s differentiated HIV service (dis)engagement. Our application of practice theory to engagement in HIV care, as experienced by HIV service users and providers in six sub-Saharan African countries, highlights the need for a practice-based approach in the delivery of differentiated and patient-centred HIV services.

## Introduction

Although varied engagement with HIV services has always been an issue in the HIV response, it has risen to the top of the list of policy priorities in the era of treatment as prevention. The HIV care continuum (also known as the HIV cascade) was initiated as a guiding model for delivering and measuring HIV services, allowing us to understand better barriers and facilitators in the progression from initial diagnosis of HIV to viral suppression.^1 2^ The continuum has helped to standardise measures of care and delivery of HIV treatment at an unprecedented scale, prolonging the lives of millions of people.^3 4^ Yet, a large proportion of unexpected deaths still occur throughout the HIV care continuum,^5 6^ and studies reveal how people living with HIV (PLHIV) are affected by social structures and step in and out of HIV services in multiple ways, leading to different forms of disruption to progression along the care continuum.^7–10^ There is a long history of scholars calling for recognition of how contextual factors, beyond biomedical interventions, shape HIV treatment experiences, engagement and outcomes.^11–15^ However, Blue and colleagues^16^ argue it can be difficult to pinpoint exactly how contextual factors interact with and affect health and how recognition of such factors can be translated into practical actions. They suggest that by shifting our attention to the organisation of social practices — the elements that shape our perceptions, interpretations and actions in daily life^17^ — we will be able to know exactly how structure and context intersect with agency. In this article, we consider the potential of ‘theories of practice’ for the study of (dis)engagement with HIV services and begin to unpack the assemblage of elements and life practices that shape HIV care and treatment (dis)engagement in sub-Saharan Africa.

### Theories of practice and (dis)engagement with HIV services

**Figure 1:**
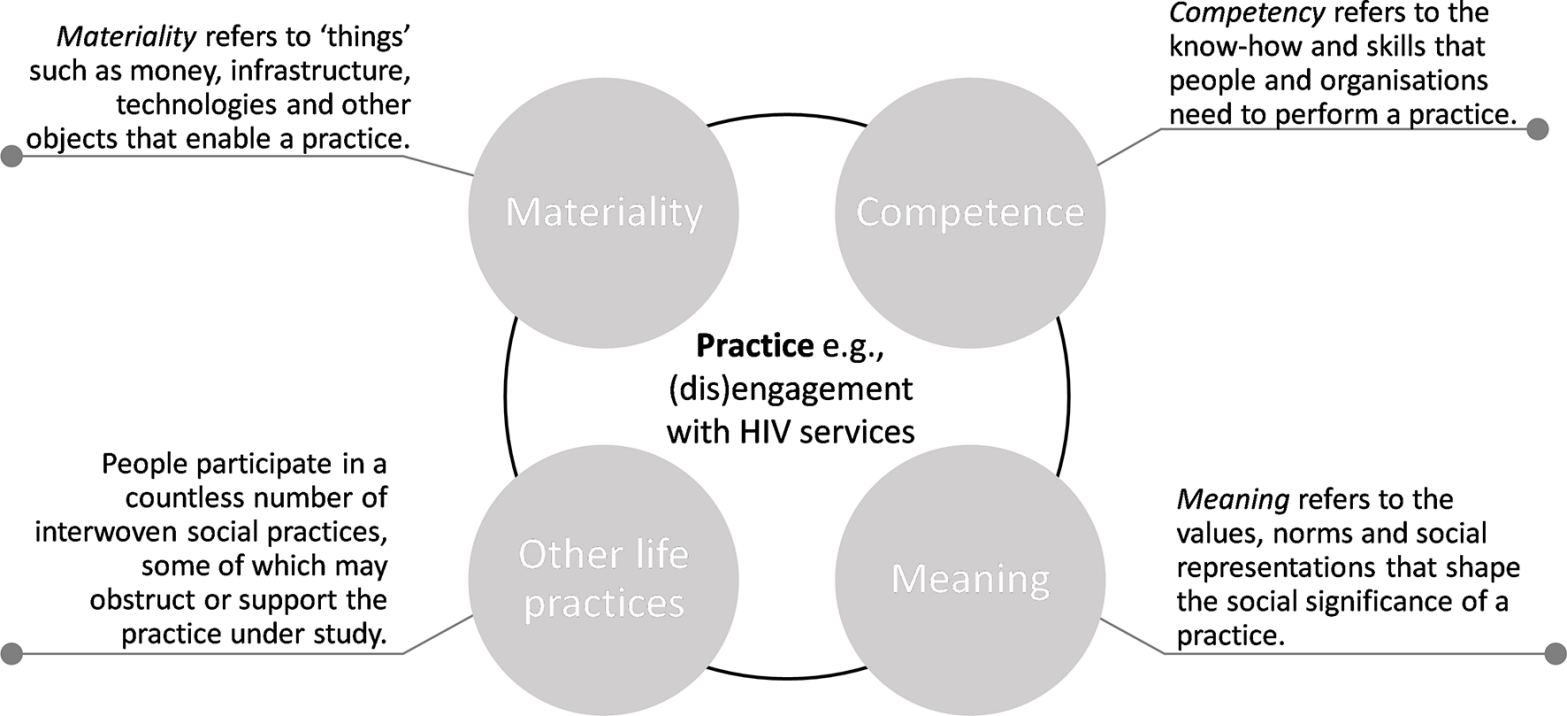
Summary of theoretical framework.

There is a no single and unified theory of practice.^18^ Instead, there is a dynamic and collegial tradition of applying and building on the work of past and contemporary theorists. Common to them all, however, is that they treat practices as the primary units of enquiry and provide conceptual tools to unpack how and why certain practices emerge, persist and disappear. Practice theorists like Nicolini,^19^ Reckwitz,^20^ Schatzki^21^ and Shove^18^ have all offered their take on the background arrangements, or elements, that condition and shape the dynamic nature of a practice, such as engaging with HIV services. Shove *et al* 18 and Blue *et al* 16 have usefully condensed these background arrangements into three elements—namely, materialities, competencies and meanings—and draw our attention to the configurations and connections between these elements and other life practices (figure 1). Here, they argue, is the potential to understand what it takes for people to join, maintain or defect from a practice.

These elements, their content, configurations and constellations, provide insight to the arrangements that exist in different spatial and temporal localities and may be able to explain  differences in practice between people and settings. This encourages us to examine how dynamically integrated elements shape the way HIV service engagements are enacted. It further helps us understand how  changes in the way HIV services are delivered, reconfigure and shape engagement with HIV care and treatment services. For instance, reducing or extending the opening hours of a health clinic (materiality, infrastructure) may change the ability of PLHIV to engage with HIV services.

Another important practice dynamic pertains to the connections and interactions between practices. People participate in a countless number of interwoven social practices, many of which coexist in harmony, while others codepend and may compete or collaborate with the practice(s) under investigation.^18 22^ Schatzki^22^ and Shove *et al* 18 speak of *bundles of practices* to describe such an assemblage of codependent practices and highlight the importance of understanding how a bundle of practices, through their connections, coevolve, share and compete for resources. This encourages us to identify the range of life practices that facilitate or inhibit engagement with HIV services and to use this insight to form or break and strengthen or weaken the links between them, with the aim of supporting HIV service engagement.

Against this background, and in support of a call from Blue *et al* 16 for more practice-oriented public health policy, we investigate (1) how engagements with HIV services are constituted and enacted by multiple elements and not just people alone and (2) how engagements with HIV services relate to other everyday practices.

## Methods

In this study, the data were obtained from a qualitative multicountry study that examines how PLHIV, in the context of their social worlds, interact with HIV services. Ethical approval was granted by the London School of Hygiene and Tropical Medicine and the relevant ethics boards at each of the study settings. Informed and written consent was obtained from all participants on the agreement that confidentiality would be assured. Pseudonyms are therefore used throughout.

### Study locations and participants

The study was conducted in Karonga (Malawi), Rakai and Kyamulibwa (Uganda), Kisesa (Tanzania), Kisumu (Kenya), Manicaland (Zimbabwe) and uMkhanyakude (South Africa)—settings that are hard hit by the HIV epidemic. We purposefully recruited a mix of healthcare workers (n=53), PLHIV (n=255) and family members of people who have died from HIV (n=48). Participants were recruited via health and demographic surveillance databases, health clinics or following verbal autopsy^23^ interviews with family members of recently deceased PLHIV to represent a broad distribution of sex, age and diagnosis and care histories (table 1).

**Table 1:** Study participants shown by sampling category and country

Country	Demographic surveillance site	Healthcare worker	People living with HIV			Family member of a deceased
			Diagnosed, not on ART	On ART*	LTFU†	
Uganda	Rakai	6	15	15	6	8
Uganda	Kyamulibwa	5	8	8	4	5
Kenya	Kisumu	8	10	15	6	11
Tanzania	Kisesa	7	13	20	4	6
Malawi	Karonga	5	9	20	4	6
Zimbabwe	Manicaland	4	16	35	8	6
South Africa	uMkhanyakude	18	1616	17	6	6
Total		53	87	130	38	48

*ART: the PLHIV could have been taking ART for variable periods of time; countries had varying cut-off points but generally captured recently initiated and then longer term (over 5 years).

†LTFU:  PLHIV had not collected ART from their registered clinic for a country-specific predetermined period of time.

ART, antiretroviral therapy; LTFU, lost to follow-up.

### Data collection and analysis

Topics guides for PLHIV sought to elicit differentiated experiences of (dis)engagement with HIV testing, care and treatment services and place these in the context of their lived realities. Healthcare workers were invited to reflect on their experiences of engaging PLHIV and offering HIV services. Interviews with family members of the deceased sought to understand the circumstances that led to their death.

Interviews were conducted in the local language of each setting between October 2015 and May 2016, either in the participant’s own homes or in a health clinic. Interviews were audio-recorded and lasted between 45 and 90 min. All interviews were anonymised and either summarised (Kyamulibwa) or transcribed (Karonga, Kisesa, Rakai, Kisumu, Manicaland, uMkhanyakude) into English. Summaries were done in Kyamulibwa due to researchers at this site being historically trained in this method. Using NVivo 10, we drew on the thematic network analysis method by Attride-Stirling^24^ to cluster codes into basic and organising themes, which formed the development of a broad analytical coding framework.^24 25^ Emerging findings and comparisons between groups and countries were discussed and contrasted in an analysis workshop attended by study coordinators from each setting. This study  draws on data coded, and thematically organised, by site coordinators under the heading 'HIV service engagement'. This subset of data was subsequently subject to a second round of thematic organising, driven by the two practice dynamics outlined above. The analysis is thus focused on engagement with HIV services as practice, rather than on characteristics of individual users of HIV services.

Additional methodological details concerning the study are given  in an online-only supplement included in the editorial paper^26^ at http://dx.doi.org/10.1136/ sextrans-2017-053172.

## Results

**Figure 2:**
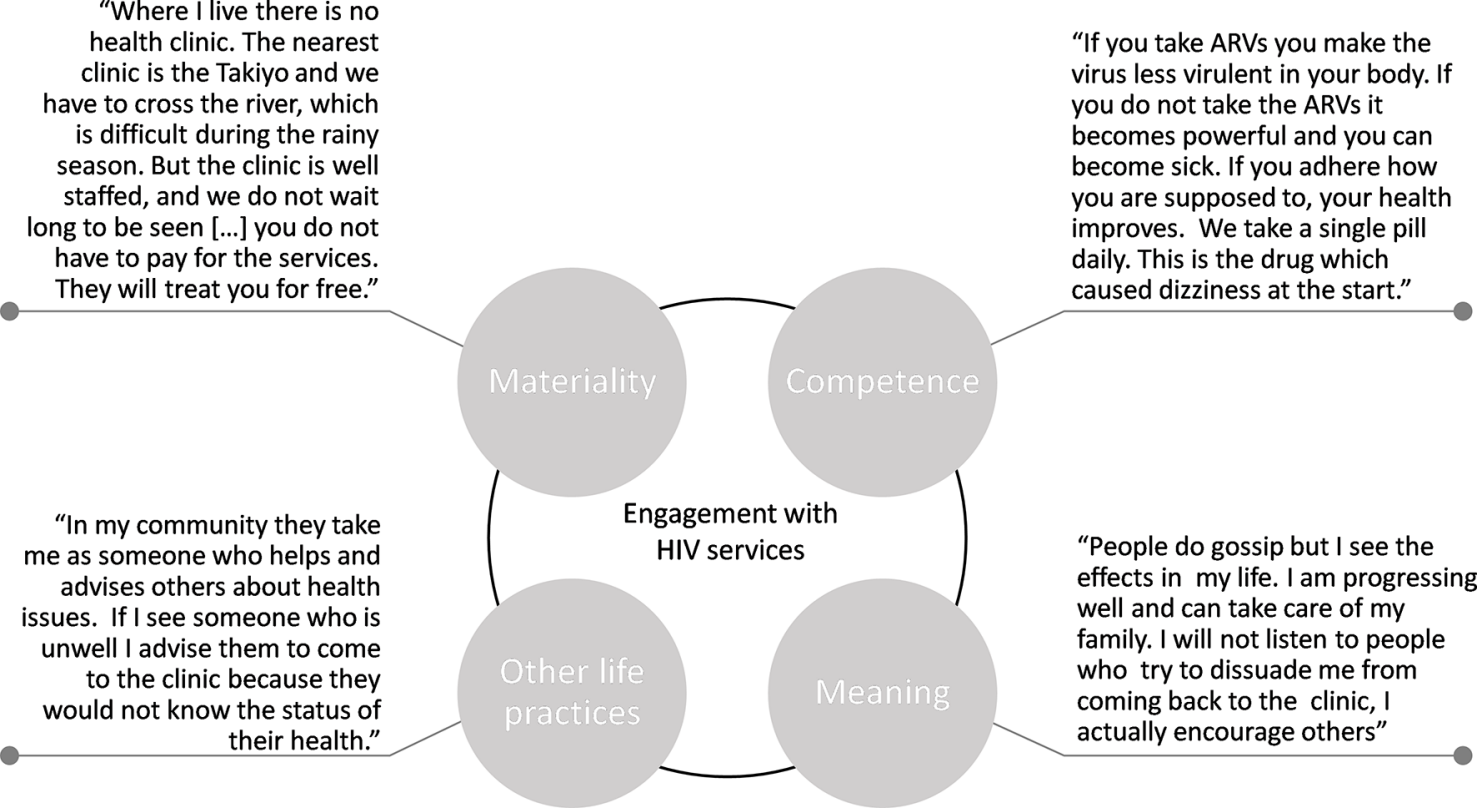
Quotations from a male on ART, Southern Africa. ARV, antiretroviral drug.

**Figure 3:**
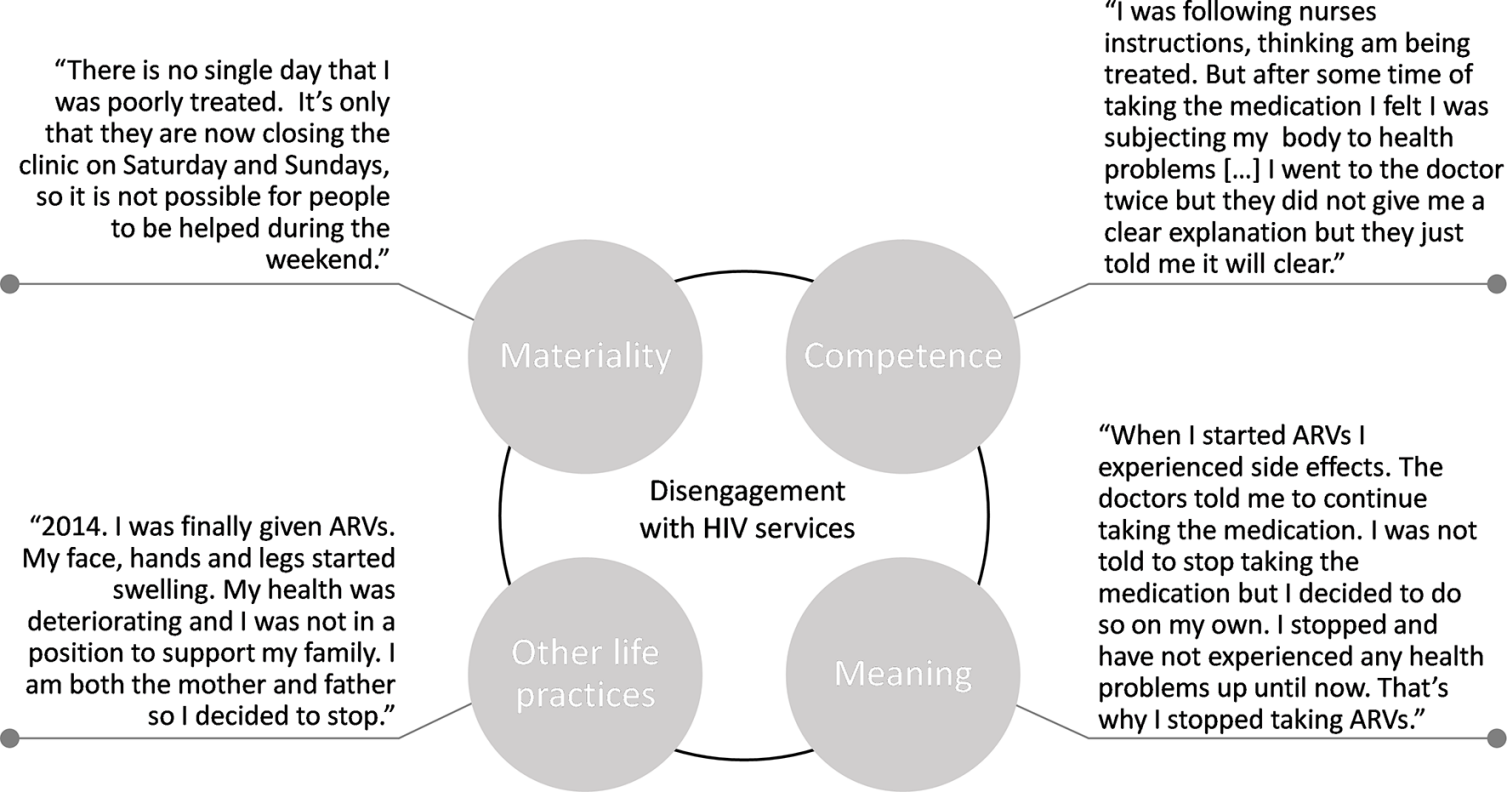
Quotations from a female lost to follow-up, Southern Africa. ARV, antiretroviral drug.

In our findings, we present both thematic and person-centred quotations (figures 2 and 3) and refer to other papers published in this special issue that report on this dataset.

### Elements of ‘engagement’ with HIV services

*The ‘materiality’ of engagement with HIV services*: In all settings, participants observed that HIV services had improved from ‘the past’, underlining the temporal and dynamic nature of HIV infrastructures. Decentralisation of HIV services, as experienced by participants in most settings, meant that some people reported having to walk shorter distances and experiencing reduced waiting times:

In the past we had problems because people had to travel long distances to access their medication but right now it can be accessed near. […] They appreciate the assistance that they get from local clinics. (*Female, on ART, Southern Africa*)

The growing availability of free drugs was also noted (see figure 2). Nonetheless, in all sites, we observed variances in engagement with HIV services because of material constraints. These included supply barriers, such as poorly resourced health infrastructures, including occasional antiretroviral therapy (ART) stock-outs, reduced opening hours (see figure 3) and distance to the clinic. A materiality quote in figure 2 alludes to how distance barriers can vary seasonally, with swarming rivers requiring people to find alternative routes to go to the clinic to collect antiretroviral drugs during the rainy season.

Furthermore, household-level poverty continues to obstruct engagement with HIV services. Poverty restricts access to nutritious foods, public transport and over-the-counter medications to cope with side effects (see Bukenya *et al* 27). In South Africa, for instance, a number of healthcare providers talked about the importance of ensuring access to food parcels or disability grants to poor and vulnerable patients. This speaks to the poverty experienced by many PLHIV and the few social welfare services available in the South African setting to support engagement with HIV services.

*The ‘competence’ of engagement with HIV services*: Healthcare providers in all settings worked under the principle that they needed to recruit PLHIV into HIV care and treatment, towards the teleological aim of ‘staying alive’. This involved equipping PLHIV with the practical knowledge required to successfully engage with HIV treatment services and manage HIV stigma. This included instructions on what to eat and drink, safe sexual practices, to whom to disclose HIV status, how to relate to a spouse and how to access and adhere to antiretroviral drugs (see Ondenge *et al* 28). A number of participants attributed the state of their current health to this practical knowledge.

What they did well for me to be where I am today is the fact that they counselled and educated me […] They continue to counsel me against negative things said about HIV positive people. (*Female, on ART, Southern Africa*)

We found that the ability of healthcare staff to impart this knowledge, and for PLHIV to take on board their knowledge and advice, was intrinsically linked to the staffing and resourcefulness of health clinics (cf. *things*) and whether PLHIV hold staff in high regards (cf. *meanings*) (see Ondenge *et al*^28^). In all sites, we also identified PLHIV who actively encouraged friends and family to go and get tested or discursively imparted treatment knowledge to peers who were at risk of defaulting:

I give advice if the person is on treatment. She is supposed to follow the procedures which she is given at the hospital and not stop taking the medicine. (*Female, on ART, Southern Africa*)

In general, family and community members appeared central to the project of building ‘HIV literacy’ and creating an enabling environment where HIV-related knowledge could be shared and encourage engagement with HIV services.

*The ‘meanings’ of engagement with HIV services*: A perceived and experienced closeness to HIV, having directly witnessed or experienced the impact of HIV and trust in the value of treatment and care, emerged as central to accepting treatment (see Renju *et al* and Wringe *et al* 29 30).

I accepted treatment because I can see they [ART] give life. In the past you would see people wasting away and die. (*Male, on ART, Southern Africa*)

Ideologies, norms and fears circulating in the communities were also found to affect people’s different abilities to engage with HIV services. In all settings, persistent stigma made it difficult for some PLHIV to accept their HIV status, affecting their ART initiation (see Bonnington *et al* 31). PLHIV on ART who experienced improved health appeared to find positive meaning from their engagement (eg, able to care for the family), helping them manage and resist stigma (see figure 2). There were also numerous accounts conveying what can be referred to as a ‘normalisation’ of HIV (see Bonnington *et al* 31). In Tanzania, for example, a woman recently diagnosed with HIV exemplifies how this normalisation of HIV makes living with, and disclosing HIV, easier for her:

When I informed him [husband] about the test, he told me not to be sad because that was a normal issue because many people are infected … when you follow up on taking your medicine you will eventually feel like others. (*Female, pre-ART, Eastern Africa*)

These brief examples begin to unpack the material things, competencies and meanings that either enable or hinder engagement with HIV services. Rather than seeing them as separate contextual factors, practice theory encourage us to explore how health services, ART and poverty (things), and the HIV know-how of healthcare workers, PLHIV and community members (competence), converge to influence how PLHIV perceive, experience (meaning) and ultimately practice (dis)engagement with HIV services (see figures 2 and 3). Our findings allude to links between various key elements, for example, how the improved availability of ART (things)—in some settings—has transformed HIV from being a fatal disease to a chronic illness (meaning) or how the resource availability of health facilities (things) influence the know-how of healthcare staff and PLHIV (competence). Recognition of these links encourages us to explore how engagement with HIV services is either amplified or attenuated by the presence or absence of particular elements. The elements are also not distributed evenly across sites and changed over time and conditioned by the discursive, cultural, political and economic fabric of a given context. Negative references to ‘the past’ and positive statements about the ‘now’ suggest that some elements have been reconfigured over time, impacting people’s experiences of, and commitment to, engagement with HIV services.

### ‘HIV service engagement’ and other life practices

Engagements with HIV services do not happen in a vacuum but are inextricably interwoven with other life practices and a bundle of supportive practices, such as disclosing HIV status, managing stigma, relationship building with health providers and participating in support groups. The practice of accepting one’s HIV status emerged as particularly influential in instigating supportive practices, such as participating in peer groups or developing health-enabling patient–provider relationships (see Ondenge *et al*^28^). Accepting or denying one’s HIV-positive status, thus, had knock-on effects on other practices associated with HIV service engagement. The configuration of this complex of supportive or conflicting practices appeared to be co-dependent on the elements described above, resulting in significant variation.

Other life practices also appeared to either compete or collaborate with engagement practices. Clients were not merely HIV service users; they were also parents, spouses, breadwinners and believers. A number of participants engaged with faith healing and alternative and complementary medical practices, and this influenced their engagement with HIV services (see Moshabela *et al* 32). Parenting could play a significant role in engagement, too. A woman from Zimbabwe made a conscious decision to stop treatment, explaining that the treatment and associated side effects prevented her from being a parent (see figure 3).

For other participants, to stay alive and to be a parent for their children served instead as a key motivator to engage with HIV services (see McLean *et al* 33). Working, too, could take precedence over going to HIV services. In Kenya, a man who was asked to come to the clinic regularly failed to show up out of fear he would lose his job:

If you are my boss you cannot allow me to leave work [to go to the clinic] every three days or once a week. (*Male, lost to follow-up, Eastern Africa*)

## Discussion

Our findings shed light on some of the ways in which social structures and contextual factors shape engagement with HIV services. We applied theories of practice to shift attention away from the characteristics of PLHIV and lists of the social determinants or ecological factors that shape engagements with HIV services, to the social organisation of HIV service engagement as practice. This revealed how the availability, absence and connections between requisite materialities, competencies and meanings shape engagement with HIV services. It also revealed that HIV service engagement, as a practice, is influenced by a host of other social practices, characterising the lives of people in low-resource and high HIV prevalence communities in sub-Saharan Africa.

While many of our observations have been noted before, recognition of their connections, or of the significance of some elements and practices, offers important clues for how engagements with HIV services become easier in some households or communities than in others. For instance, we noted that well-managed, drug-filled and accessible HIV services (materiality that is considered meaningful), discursive and practical knowledge through counselling (competency) and embodied experiences of HIV (meaning), such as improved health, are ‘resources’ for engagement with HIV services. If one of these elements is removed, for example, if drugs are unavailable or if a client feels healthy and cannot comprehend seroconversion in absence of symptoms, engagement with HIV services may get disrupted. Meaning emerged as particularly noteworthy, with PLHIV projecting meaning to the ‘things’ or ‘discursive knowledge’ they encountered.

We noted temporal differences in some settings where recent improvements in HIV service delivery,^4^ coupled with a discursive normalisation of HIV, have led to a reconfiguration of elements, which enhances people’s experiences of, and enables engagement with, HIV services. This, however, is not sufficient to sustain engagement. We also noted that engagement is affected by a bundle of related practices, such as accepting and disclosing one’s HIV status, and recurrent enactments, such as being a parent or a spouse. Depending on the context, being a parent or a spouse can either sustain or disrupt engagement.^34^ This illustrates that HIV service engagement, as practice, must be considered in relation to other social practices. We believe these findings may offer some explanation to high rates of loss to follow-up^7 9 35 36^ and mortality^5 6^ along the HIV care continuum. Others who have offered related critique have focused on the therapeutic itineraries or illness trajectories of PLHIV, demonstrating how particular life circumstances impact their ability to manoeuvre HIV treatment and care engagement.^37 38^ However, by shifting the analytical focus from individuals to the elements and practices that shape individuals’ engagement with HIV services, theories of practice offer a new and innovative framework for understanding HIV service engagements, while simultaneously transcending the dualisms of individual agency and structural conditions^18^ that guide much current HIV treatment literature. Although we have alluded to some country differences here (eg, welfare services in South Africa), future research needs to disentangle how different cultural, socioeconomic and policy spaces within and between countries shape engagement with HIV services. Nonetheless, we have provided a brief snapshot of what theories of practice can offer the study of HIV services (dis)engagement. Although the theory is not without its critics,^39 40^ we encourage other researchers to use the analytical tools offered by this approach to study and improve engagement with HIV services and health services more generally.

## Conclusion

HIV service engagements should be positioned within a constellation of practices at the local level, many of which will not ‘shift’ easily to accommodate what is needed to perform engagement. Standardised expectations of patient engagement with HIV services, if not negotiated among other practices locally and not only individually, can run counter to its actualisation. The often poor fit between HIV care and treatment services and the lived realities of PLHIV call for a broader practice-oriented HIV response. This could involve a mix of structural initiatives and more patient-centred and differentiated HIV care and treatment services, which, when combined, can tweak and address the elements and bundles of practice that shape (dis)engagement with HIV services.

**Key messages**

- People living with HIV are affected by numerous factors and step in and out of HIV services, leading to different forms of disruption to progression along the HIV care continuum.
- It is difficult to pinpoint how social structures interact and translate into varied engagements with HIV services.
- Theories of practice provide HIV researchers and practitioners with a useful vocabulary and analytical tools to understand and steer differentiated HIV service (dis)engagements.
- Standardised expectations of patient engagement with HIV services, if not negotiated among other practices locally, and not only individually, can run counter to their actualisation.
